# The economic burden of diarrhea in children under 5 years in Bangladesh

**DOI:** 10.1016/j.ijid.2021.04.038

**Published:** 2021-06

**Authors:** Md. Zahid Hasan, Gazi Golam Mehdi, Gatien De Broucker, Sayem Ahmed, Md. Wazed Ali, Jorge Martin Del Campo, Dagna Constenla, Bryan Patenaude, Md. Jasim Uddin

**Affiliations:** a International Centre for Diarrhoeal Disease Research, Bangladesh (icddr,b); b International Vaccine Access Center, Johns Hopkins Bloomberg School of Public Health, Baltimore, MD, United States; c Mathematical Modelling Group, Oxford University Clinical Research Unit (OUCRU), Ho Chi Minh City, Vietnam; d Centre for Tropical Medicine and Global Health, Nuffield Department of Medicine, University of Oxford, United Kingdom; e Department of Tropical Disease Biology, Liverpool School of Tropical Medicine, Liverpool, UK; f GlaxoSmithKline Plc., Panama City, Panama; g Department of International Health, Johns Hopkins Bloomberg School of Public Health, Baltimore, MD, United States

**Keywords:** Diarrhea, Gastroenteritis, Cost of illness, Bangladesh, Vaccine-preventable disease, Economic burden

## Abstract

**Background:**

Diarrhea is a leading cause of morbidity and mortality among under-five children in Bangladesh. Hospitalization for diarrhea can pose a significant burden on households and health systems. The aim of this study was to estimate the cost of illness due to diarrhea from the healthcare facility, caregiver, and societal perspectives in Bangladesh.

**Method:**

A cross-sectional study with an ingredient-based costing approach was conducted in 48 healthcare facilities in Bangladesh. In total, 899 caregivers of under-five children with diarrhea were interviewed face-to-face between August 2017 and May 2018, followed up over phone after 7–14 days of discharge, to capture all expenses and time costs related to the entire episode of diarrhea.

**Results:**

The average cost per episode for caregivers was US$62, with $29 direct and $34 indirect costs. From the societal perspective, average cost per episode of diarrhea was $71. In 2018, an estimated $79 million of economic costs were incurred for treating diarrhea in Bangladesh. Using 10% of income as threshold, over 46% of interviewed households faced catastrophic expenditure from diarrheal disease.

**Conclusion:**

The economic costs incurred by caregivers for treating per-episode of diarrhea was around 4% of the annual national gross domestic product per-capita. Investment in vaccination can help to reduce the prevalence of diarrheal diseases and avert this public health burden.

## Highlights

• Each episode of diarrhea incurred $29 direct and $34 indirect costs to caregivers.
• The highest costs of $136 per child were incurred at private healthcare facilities.
• Around 46% of caregivers experienced catastrophic healthcare expenditure resulting from diarrheal treatment.
• In 2018, an estimated 4% of GDP per capita was spent on diarrheal treatment.

## Background

Diarrhea is identified as one of the leading causes of infectious deaths worldwide among children under five, resulting in an estimated 526 000 deaths per year or nearly 1500 childhood deaths each day (Liu et al., 2016, Schroder et al., 2019). In low- and middle-income countries (LMICs), diarrhea is the most common cause of childhood illness and healthcare visits, especially in South Asia. (Black et al., 2008). As in many other LMICs, diarrhea is one of the major public health problems in Bangladesh, with persistent diarrhea prevalent throughout the year.

Diarrhea is defined as a condition that involves unusually frequent and liquid bowel movements; more than three or more times in a 24-h period. The disease can be characterized as ‘acute watery’ if it lasts less than 14 days, ‘persistent’ if it lasts more than 14 days, and ‘invasive’ if caused by infection due to pathogens (WHO, 2002, Alam and Ashraf, 2003). Unsafe water, poor sanitation and hygiene, and malnutrition are major risk factors for childhood diarrheal disease (WHO, 2014). Studies have shown that diarrheal disease is associated with episodes of flooding, contaminated drinking water, poor personal hygiene, and socioeconomic factors (Hoque et al., 1996, Sack et al., 2006, Wu et al., 2011a, Wu et al., 2011b). Moreover, extreme weather factors, such as higher temperatures and rainfall, increase the incidence of childhood diarrhea (Wu et al., 2014).

In 2018 around 1 122 681 episodes of diarrhea cases were reported in different levels of healthcare facility in Bangladesh (DGHS, 2020). Other than mortality and morbidity, diarrhea can also have a catastrophic economic impact on households due to out-of-pocket (OOP) payments for healthcare. These economic impacts disproportionately burden the poorest households compared with the richest (Khan et al., 2017).

Several types of intervention are available for the treatment of diarrheal disease. These include oral rehydration solutions, zinc treatment, continued feeding, and antibiotic treatment for certain strains of diarrhea (cholera, shigella, and cryptosporidiosis). These diarrheal treatments are available in all types of health facility in the Bangladesh health system, which comprizes public providers, private for-profit (PFP) providers, and private not-for-profit (PNFP) providers. The public health facilities under the Directorate General of Health Services (DGHS) is divided into different tiers: national, divisional, district, upazila (sub-district), union/ward, and community. People have almost free access to these facilities. The PFP health sector includes large and small hospitals, medical practitioners (i.e., doctors and individual providers), and informal healthcare providers. The PNFP providers mostly focus on maternal, child, and adolescent healthcare services in Bangladesh. These services are offered in both rural and urban areas, and are often free for the poor population. In addition to this, a large number of patients seek care from non-professional providers (Chowdhury et al., 2015).

According to WHO, immunization through vaccination can prevent a number of diseases, most of which can have serious complications (WHO 2012). Since 1979, Bangladesh has initiated the Expanded Program on Immunization (EPI) as an integral part of Bangladesh’s efforts to reduce child mortality due to vaccine-preventable diseases. Most of the vaccines included in the EPI are funded by the Global Alliance for Vaccines and Immunizations (Gavi) (DGHS, 2015). Although vaccines are globally regarded as one of the most cost-effective public health interventions, gaps exist in the evidence base relating to their broader economic impact, including the cost of illness averted due to vaccination in LMICs. There is also an overall lack of guidelines on best practices for primary data collection in LMICs relating to cost-of-illness analyses for major childhood diseases.

A number of studies have been conducted in many other LMICs on the cost of illness of diarrheal disease (Mendelsohn et al., 2008, Rheingans et al., 2012a, Burke et al., 2014). However, limited studies are available that capture the total costs of diarrheal episodes at different levels of the health system. Such studies have an important role in policy making and evidence generation for international comparisons. Despite several existing studies on the economic impact of diarrheal disease in Bangladesh (Rheingans et al., 2012b, Sarker et al., 2013, Sarker et al., 2018, Sarker et al., 2019, Das et al., 2015), none of them captured information from all relevant levels of the health system, and few estimated the aggregate societal costs of diarrheal disease and the impact that diarrheal disease had on households.

Recently, Bangladesh’s income status classification was redefined from low-income to LMIC by the World Bank (The World Bank Group, 2018), which will accelerate the country’s transition out of Gavi’s donor support in providing necessary immunization services. It is vital to understand the economic impact conferred by diarrhea vaccination and thereby provide evidence to support ongoing investment in vaccination programs. In this context, the cost-of-illness studies of childhood diarrhea can provide critical information to stakeholders for use in evidence-based financial planning and decision making.

Evidence on the cost of illness of diarrhea generated through this study will be useful in conducting the cost-effectiveness analysis of diarrheal interventions in future, which is an important criterion for assessing prevention programs in Bangladesh. Thus, the broad objective of this study was to estimate the cost of illness due to diarrhea from the healthcare facility, caregiver, and societal perspectives in Bangladesh, and to examine how the OOP spending relating to diarrhea leads to catastrophic healthcare expenditure (CHE) among households in which children under five are suffering from diarrheal disease. The specific objectives are as follows:

i To estimate the cost drivers of diarrheal disease.
ii To estimate the average economic cost (both financial and indirect) of treating diarrhea among the different types of health facility.
iii To estimate the impact of OOP spending resulting from diarrheal disease on CHE for households whose children under five are suffering from diarrhea.

## Methods

### Study design

Our study involved an incidence-based cross-sectional study design, paired with an ingredient-based costing approach in order to capture the costs associated with a full episode of diarrheal disease. The cost of treating diarrhea beyond 14 days after the initial discharge was not assessed.

### Study population and sites

**Figure 1:**
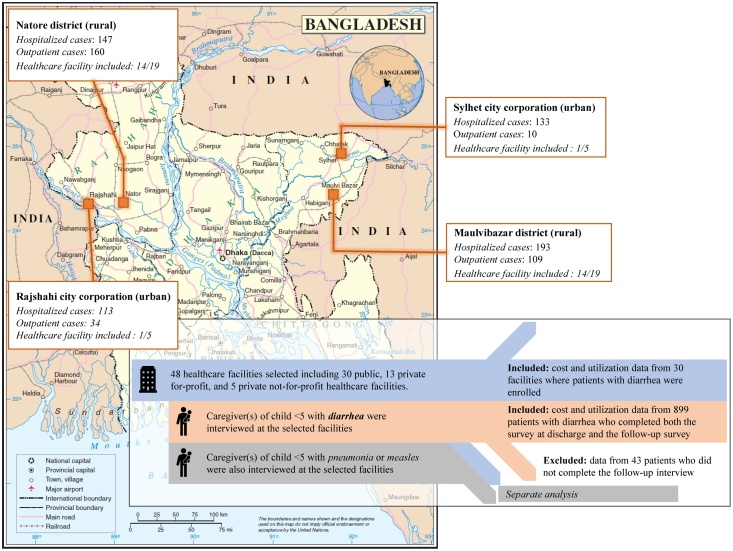
Map of the study sites; based on the United Nations Map No. 3711, Rev. 2, January 2004.

In order to better represent overall immunization coverage in Bangladesh, we purposively selected a low-performing division, Sylhet (61.1%), and a high-performing division, Rajshahi (83.6%), as study sites (DGHS, 2013). For each of the two selected divisions, one rural district and one city corporation (each division has one city corporation) were selected. Within each selected district or city corporation there were different health facility tiers (e.g., primary level facility with or without inpatient care, secondary level facility, and tertiary level facility) with different types of ownership (e.g., public, PFP, and PNFP). In Moulvibazar district of Sylhet division there are 222 public, 13 PFP, and 17 PNFP facilities and in Natore district of Rajshahi division there are 224 public, 22 PFP, and 14 PNFP facilities. Of the two city corporations, there are three public, 34 PFP, and 23 PNFP facilities in Sylhet city corporation and eight public, 63 PFP, and 39 PNFP facilities in Rajshahi city corporation (MOHFW, 2020). To capture at least one facility from each type of ownership and one facility from each level, 19 facilities from each district and five from each city corporation (24 from each division) were selected (Figure 1). These included 30 public and 18 PFP and PNFP facilities. The facilities were selected according to the number of measles, pneumonia, and diarrhea cases reported for the prior year (2015–16).

For the caregiver’s perspective, adult caregiver(s) (aged over 18 years) of the selected children (aged 0–59 months) were included. The target patients in each facility were identified through clinical assessment and were not necessarily confirmed by laboratory tests. Cases with comorbidities were excluded.

For collecting data on facility-level costs from the provider perspective, administrators and managers of public healthcare facilities, along with medical staff, laboratory technicians, statisticians, and storekeepers, were recruited.

### Data collection

Interviews were carried out with administrators and managers of the public healthcare facilities to collect cost data from the provider perspective, and with medical staff, laboratory technicians, statisticians, and storekeepers for data on healthcare utilization from August 2017 to May 2018. Administrative records were used to support or adjust estimates when required. Data were collected from the pediatric ward only for cases in tertiary- and secondary-level hospitals.

Two healthcare facility surveys were conducted. From the first survey, data on the costs of different medical items and the average time spent by different healthcare professionals on diarrhea cases were collected. The second was a monthly follow-up facility survey, which captured the overhead costs from annual expenses and utilization rates for supplies and medications from inventories. Additional data on medication pricing in the private sector were collected via interviews with pharmacy owners or pharmacists in charge of selected pharmacies.

For hospitalized cases, caregivers were interviewed during discharge from the facility and followed up by phone within 7–14 days. For outpatient cases, caregivers of sick children were interviewed as they were exiting the facility. During these interviews, information was obtained about OOP payments for the treatment of their child’s diarrhea before being admitted to the hospital or outpatient clinic, while in the hospital or outpatient clinic, and after being discharged from the hospital or outpatient clinic. These included payments for their child’s hospital stay, medications, tests, and procedures, as well as transportation costs, and the costs of meals and lodging relating to their child’s illness. Information on time devoted to providing care for the children (productivity loss) while in the hospital or outpatient clinic and post discharge was also obtained. Different costs items are presented in Table 1. Finally, information was collected on the characteristics of the respondents’ households, household expenditures, and income of the members to assess their socioeconomic status and the impact that their child’s illness was having on the household.

**Table 1:** Summary table of cost items.

Types of cost	Included cost items	Types of survey
Household direct medical (costs incurred as a result of medical treatment)	Consultation fee Hospital costs/bed fee Medicine Diagnostic	Patient caregiver survey and follow-up survey
Household direct non-medical (costs incurred for medical management but not associated with treatment)	Food Transportation Accommodation of caregiver Any informal payments	Patient caregiver survey and follow-up survey
Household indirect (costs incurred not for medical management of the disease but through losses such as lost wages)	Wage losses for the patient caregiver due to care of the patients	Patient caregiver survey and follow-up survey
Provider (costs incurred by the provider for providing treatment to the patients)	Capital items Overheads (electricity, telephone) Salary of staff Supplies Medicines	Health facility main (one-time) and monthly surveys

### Costing methods

Costs were reported in 2018 US$, using a conversion rate of 1 USD = 83.5 Bangladeshi takas (BDT) (The World Bank, 2019). Cost categories were defined according to Jo (2014).

All costs from the public provider perspective were patient-specific with the exception of overhead, labor, and capital costs. The latter items were estimated as shared costs on the basis of patients in a pediatric ward. The capital costs items were annualized over their useful lifetime at a 3% discount rate. A lifetime of 50 years for infrastructure and 5 years for medical equipment was assumed (Hoque et al., 2011). The costs for overhead, labor, and capital consumed to treat an episode of diarrhea were estimated on the basis of patient-days (see Eq. (1)). For outpatient cases, the estimated costs for a day of inpatient care were used.

(1) $$S=\overset{\overset{n}{m}}{\underset{\underset{j=0}{i=0}}{\Sigma }}\frac{c_{j}\times los_{i,j}}{p_{j}}$$

In Eq. (1), *S* is the total cost attributable to diarrhea, *c* the total annual cost for overheads, labor, and capital, *p* is the number of patients using the health facility in 1 year (with *los_i_* the length of stay in days for caregiver *i* over *n* total caregivers) and for healthcare facility *j* over *m* total facilities.

However, in some facilities there were wards dedicated to diarrhea treatment. In these cases, the capital costs associated with each ward were calculated according to utilization of the ward by diarrhea cases only. If the capital item was shared across the whole pediatric ward, in these cases the capital costs were calculated based on the utilization rate of the whole pediatric ward. Patient-specific utilization was considered for combining all other costs.

All direct costs of the caregivers were itemized. Indirect costs were estimated using a human capital approach, considering the average income of the household head and the time spent getting to/from the healthcare facility, in the healthcare facility, and providing care at home after discharge. Additionally, the detailed time losses for the caregivers, along with their indirect costs, were reported.

A one-way ANOVA was applied for testing the variances of different types of cost for treating an episode of diarrhea (e.g., direct, indirect, and total costs) by the child’s gender, the household’s residence (urban or rural), the type of visit (inpatient or outpatient), the type of facility (public, PFP, or PNFP), and the length of stay. Based on the one-way ANOVA, if the assumption of equal variance was not supported, Wilcoxon–Mann–Whitney tests were applied for independent variables with two categories and Kruskal–Wallis tests for the independent variables with more than two categories. If the assumption of equal variance was supported, *t*-tests were applied.

Societal costs were estimated by combining caregiver costs and provider costs for each illness. Private facilities are not subsidized and charge all the costs to the caregivers through fees and copayments. Provider costs were only applicable for public facilities because these facilities are subsidized by the government. However, for estimating the societal costs, the costs incurred by the caregivers and those incurred by the public providers were combined. The country-level costs for diarrhea were estimated by multiplying the societal costs per episode by the national incidence of diarrhea cases for 2018.

### Principal component analysis

Principal component analysis (PCA) was applied to classify the households into different categories of socioeconomic status, based on the household’s dwelling characteristics and the possession of durable assets. The PCA scores were generated using household dwelling characteristics (e.g., roof, wall, and floor materials, sources of water, types of sanitation use, and utilities) and the possession of durable goods (e.g., mobile phones and televisions) (Vyas and Kumaranayake, 2006). Using the scores generated from PCA, the households were divided into asset quintiles.

### Catastrophic health expenditures

The proportion of households that faced CHE from diarrheal treatment was estimated using several definitions of CHE (Pradhan and Prescott, 2002, Wagstaff and van Doorslaer, 2003, Xu et al., 2003, Xu et al., 2006, Russell, 2004). If direct OOP expenditure (direct medical and direct non-medical) for an episode of diarrhea exceeded a certain threshold of monthly income of the household head or the monthly household expenditures, the household was said to face CHE. The total monthly household expenditure comprized both food expenditure and non-food expenditure. We classified A household was classified as experiencing CHE related to an episode of diarrhea when OOP expenditure for the episode exceeded a certain threshold. Two such thresholds were applied for determining the incidence of CHE: i) 10% or 25% of total household expenditure, as used by Wagstaff and van Doorslaer (2003); and ii) 40% of non-food consumption expenditure as a proxy of the household’s capacity to pay, as used by O’Donnell et al. (2008) and Xu et al. (2003).

## Results

In total, 899 diarrhea episodes were captured during the data collection period, of which 586 were inpatient cases (65%) and 313 were outpatient cases (35%). Most children were under the age of 2 years (766 children, 85%) and most were male (577, 62%). Over 79% of the caregivers were women. A majority of caregivers were living in rural areas (678, 75%). The majority of the patients had a length of stay of less than 5 days (510, 87.0%). The majority of the cases were from public healthcare facilities (548 children, 61%) followed by private for-profit facilities (348, 39%). Only three children (<1%) were recruited in private not-for-profit facilities (Table 2).

**Table 2:** Sample characteristics of diarrhea patients and caregiver of patients in Bangladesh.

Characteristics	Diarrhea cases (*n* = 899)		Characteristics	Diarrhea cases (*n* = 899)	
	*n*	%		*n*	%
Age-group			Asset quintiles		
0–5 months	104	12%	Poorest	180	20%
6–11 months	258	28%	2nd	181	20%
12–24 months	404	45%	3rd	179	20%
More than 24 months	133	15%	4th	180	20%
Gender of the child			Richest	179	20%
Female	322	36%	District		
Male	577	64%	Maulvibazar district (rural)	302	34%
Gender of the caregiver			Natore district (rural)	307	34%
Female	713	79%	Rajshahi city corporation (urban)	147	16%
Male	186	21%	Sylhet city corporation (urban)	143	16%
Education of the caregiver			Type of service		
No education	26	3%	Inpatient service	586	65%
Primary incomplete	80	9%	Outpatient service	313	35%
Primary complete	191	21%	Length of stay at IPD		
Secondary incomplete	321	36%	Up to 4 days	510	87%
Secondary complete	281	31%	5 days or more	76	13%
Residence location			Type of facilities		
Rural	678	75%	Public	548	61%
Urban	221	25%	Private for-profit	348	39%
Household size (excluding sick child)			Private not-for-profit	3	<1%
1–3 persons	133	15%			
4–5 persons	413	46%			
6 persons or more	353	39%			

### Provider costs of treatment

Table 3 shows the provider costs of treatment in public facilities. Overall, the government facilities spent about $23 for a hospitalized case and about $7 for an outpatient case on providing care. Primary healthcare facilities had an average total cost of $9 for outpatient care and $24 for a hospitalized case of diarrhea. Secondary and tertiary healthcare facilities had average total costs of $8 and $9 to treat an outpatient case and $16 and $27 for a hospitalized case, respectively.

**Table 3:** Government costs for an episode of diarrhea in 2018 US dollars by types of care at different levels of facility.

Cost	Inpatient cases				Outpatient cases			
	*Primary healthcare facilities without inpatient capabilities (n = 14)*^a^							
	Mean	SE	95% CI		Mean	SE	95% CI	
Capital					0.53	0.38	0.30	0.74
Overhead					0.00	0.00	0.00	0.00
Labor					2.46	1.83	1.40	3.51
Supplies					0.00	0.00	0.00	0.00
Medications					0.00	0.00	0.00	0.00
Total cost					4.05	0.78	2.37	5.71

	*Primary healthcare facilities with inpatient capabilities (n = 12)*							
Capital	0.63	0.14	0.31	0.96	0.24	0.05	0.12	0.36
Overhead	0.47	0.04	0.38	0.54	0.18	0.01	0.14	0.20
Labor	4.35	0.49	3.25	5.45	1.63	0.18	1.22	2.04
Supplies	0.04	0.01	0.01	0.06	0.01	0.00	0.00	0.02
Medications	16.79	3.16	9.63	23.94	6.29	1.19	3.60	8.97
Total cost	24.48	3.47	16.61	32.34	9.17	1.31	6.23	12.11

	*Secondary healthcare facility (n = 2)*							
Capital	0.23	0.04	0.11	0.34	0.11	0.01	0.06	0.17
Overhead	1.35	0.53	−0.32	3.04	0.66	0.25	−0.16	1.46
Labor	3.04	0.71	0.77	5.31	1.46	0.35	0.37	2.55
Supplies	0.10	0.07	−0.12	0.31	0.05	0.04	−0.06	0.14
Medications	7.80	4.63	−6.95	22.54	3.75	2.23	−3.34	10.84
Total cost	15.68	3.99	2.97	28.37	7.53	1.92	1.43	13.64

	*Tertiary healthcare facility (n = 2)*							
Capital	2.04	0.28	−1.50	5.56	0.66	0.08	−0.48	1.78
Overhead	0.38	0.10	−0.81	1.58	0.12	0.04	−0.26	0.50
Labor	8.24	2.49	−23.34	39.82	2.65	0.80	−7.51	12.80
Supplies	0.59	0.42	−4.81	5.98	0.19	0.13	−1.54	1.92
Medications	11.20	1.38	−6.32	28.71	3.60	0.44	−2.04	9.23
Total cost	27.27	1.23	11.59	42.95	8.77	0.40	3.72	13.81
	All facilities with IPD (*n* = 16)				All facilities with OPD (*n* = 30)			
Capital	0.71	0.16	0.36	1.06	0.38	0.06	0.26	0.50
Overhead	0.68	0.16	0.34	1.01	0.16	0.05	0.06	0.26
Labor	4.51	0.57	3.30	5.71	2.06	0.25	1.54	2.58
Supplies	0.12	0.06	−0.01	0.25	0.02	0.01	0.00	0.05
Medications	13.84	2.42	8.68	19.00	2.84	0.70	1.41	4.26
Total cost	22.63	2.54	17.21	28.05	6.53	0.74	5.02	8.05

a These healthcare facilities did not have the capacity to provide treatment for the inpatients.

In all facility levels, the main drivers of facility costs were those reported for medication and labor, for both inpatients and outpatients.

### Caregiver cost of treatment

Table 4 shows that for inpatient care, caregivers spent less in public healthcare facilities than in PFP and PNFP facilities. On average, for a hospitalized case of diarrhea in a public facility, $47 was incurred by caregivers, of which $17 comprized OOP payments. Caregivers using PFP and PNFP facilities for hospitalization incurred average total costs of $134 and $116, including $66 and $63 in OOP expenses, respectively.

**Table 4:** Total household costs for a hospitalized episode of diarrhea in 2018 US dollars by types of healthcare facilities.

Timing	Types of cost/time loss	Public healthcare facilities (*n* = 302)					Private for-profit healthcare facilities (*n* = 283)				
		Mean	SD	95% CI		*n* (*c* > 0)	Mean	SD	95% CI		*n* (*c* > 0)
Before current visit^a^	Direct medical	2.32	3.08	1.98	2.67	214	3.77	4.28	3.27	4.26	230
	Direct non-medical	0.14	0.71	0.06	0.23	31	0.80	2.79	0.48	1.13	63
	Indirect	9.93	9.69	8.83	11.03	302	19.08	17.70	17.01	21.15	283
	*Time loss [days]*	0.50	0.44	0.45	0.55	302	0.63	0.37	0.59	0.67	283
Current visit	Direct medical	2.44	2.46	2.17	2.73	302	34.05	22.95	31.37	36.74	283
	Direct non-medical	9.65	7.68	8.79	10.53	302	24.62	26.86	21.47	27.76	283
	Indirect	20.01	16.04	18.19	21.82	302	50.75	88.48	40.41	61.11	283
	*Time loss [days]*	0.98	0.58	0.91	1.05	302	1.57	1.16	1.43	1.71	283
Follow-up^a^	Direct medical	2.14	2.06	1.90	2.37	251	2.40	2.77	2.07	2.72	215
	Direct non-medical	0.38	2.87	0.06	0.71	20	0.05	0.28	0.01	0.07	15
	Indirect	0.56	3.88	0.13	1.01	22	0.16	2.41	−0.12	0.44	7
	*Time loss [days]*	0.03	0.18	0.01	0.05	22	0.00	0.01	0.00	0.00	7
Total direct (financial) cost		17.10	10.24	15.94	18.26	**302**	65.68	44.72	60.44	70.91	**283**
Total economic cost		47.47	32.06	43.84	51.10	**302**	135.60	129.98	120.40	150.81	**283**

Timing	Types of cost/time loss	Private not-for-profit healthcare facilities (*n* = 1)					All healthcare facilities (*n* = 586)				
		Mean	SD	95% CI		*n* (*c* > 0)	Mean	SD	95% CI		*n* (*c* > 0)
Before current visit^a^	Direct medical	0.00^b^	–	–	–	0	3.02	3.77	2.71	3.32	444
	Direct non-medical	0.00	–	–	–	0	0.46	2.02	0.30	0.62	94
	Indirect	21.56	–	–	–	1	14.40	14.86	13.17	15.60	586
	*Time loss [days]*	1.00	–	–	–	1	0.56	0.41	0.53	0.60	586
Current visit	Direct medical	45.15	–	–	–	1	17.78	22.53	15.95	19.62	586
	Direct non-medical	17.96	–	–	–	1	16.90	20.84	15.21	18.59	586
	Indirect	30.87	–	–	–	1	34.87	64.36	29.65	40.10	586
	*Time loss [days]*	1.43	–	–	–	1	1.27	0.95	1.19	1.34	586
Follow-up^a^	Direct medical	0.00	–	–	–	0	2.26	2.43	2.06	2.46	466
	Direct non-medical	0.00	–	–	–	0	0.22	2.08	0.05	0.38	35
	Indirect	0.00	–	–	–	0	0.37	3.25	0.11	0.63	29
	*Time loss [days]*	0	–	–	–	0	0.02	0.13	0.01	0.03	29
Total direct (financial) cost		**63.11**	–	–	–	**1**	**40.63**	**40.10**	**37.38**	**43.89**	**586**
Total economic cost		**115.54**	–	–	–	**1**	**90.16**	**103.02**	**81.80**	**98.50**	**586**

Notes:SD, standard deviation; *n* (*c* > 0), number of caregivers with costs over zero takas.

a Includes costs incurred by public and private healthcare facilities and providers.

b Respondent did not report any specific types of cost.

For outpatient cases, caregivers using public and PNFP facilities spent, on average, about $7 and $3, respectively, including $4 and $2 in OOP expenses. Caregivers using PFP facilities spent over $24, including $16 in OOP expenses (Table 5).

**Table 5:** Total household costs for an outpatient episode of diarrhea in 2018 US dollars by types of healthcare facility.

Timing	Types of cost/time loss	Public healthcare facilities (*n* = 246)					Private for-profit healthcare facilities (*n* = 65)				
		Mean	SD	95% CI		*n* (*c* > 0)	Mean	SD	95% CI		*n* (*c* > 0)
Before current visit^a^	Direct medical	0.50	1.28	0.35	0.66	92	1.52	2.13	0.99	2.05	37
	Direct non-medical	0.01	0.07	0.00	0.02	5	0.08	0.46	−0.02	0.20	6
	Indirect	0.62	1.60	0.42	0.83	246	2.00	12.10	−0.99	4.99	65
	*Time loss [days]*	0.03	0.08	0.02	0.04	246	0.06	0.29	−0.02	0.13	65
Current visit	Direct medical	0.07	0.35	0.04	0.12	167	4.78	6.00	3.29	6.26	64
	Direct non-medical	1.05	1.90	0.81	1.29	210	3.14	9.07	0.89	5.38	63
	Indirect	1.98	3.90	1.49	2.47	246	5.64	18.06	1.16	10.11	65
	*Time loss [days]*	0.09	0.16	0.07	0.11	246	0.19	0.45	0.07	0.30	65
Follow-up^a^	Direct medical	1.78	1.99	1.53	2.04	197	4.68	4.13	3.66	5.71	61
	Direct non-medical	0.12	1.05	−0.01	0.25	11	0.63	1.99	0.13	1.13	11
	Indirect	0.37	4.60	−0.20	0.95	16	1.32	5.45	−0.04	2.67	9
	*Time loss [days]*	0.02	0.18	0.00	0.04	16	0.02	0.09	0.00	0.04	9
Total direct (financial) cost		**3.56**	**3.87**	**3.07**	**4.04**	**246**	**14.83**	**15.66**	**10.95**	**18.71**	**65**
Total economic cost		**6.53**	**10.37**	**5.22**	**7.83**	**246**	**23.77**	**44.18**	**12.83**	**34.73**	**65**

Timing	Types of cost/time loss	Private not-for-profit healthcare facilities (*n* = 2)					All healthcare facilities (*n* = 313)				
		Mean	SD	95% CI		*n* (*c* > 0)	Mean	SD	95% CI		*n* (*c* > 0)
Before current visit^a^	Direct medical	0.06	0.08	−0.71	0.83	2	0.71	1.54	0.54	0.89	131
	Direct non-medical	0.00				0	0.02	0.22	0.00	0.05	11
	Indirect	0.48	0.37	−2.89	3.86	2	0.91	5.69	0.28	1.54	313
	*Time loss [days]*	0.03	0.01	−0.08	0.14	2	0.04	0.15	0.02	0.05	313
Current visit	Direct medical	0.24	0.00	0.24	0.24	1	1.05	3.34	0.68	1.43	232
	Direct non-medical	0.69	0.05	0.31	1.07	2	1.49	4.51	0.98	1.99	275
	Indirect	1.26	0.59	−4.01	6.53	2	2.73	9.01	1.72	3.74	313
	*Time loss [days]*	0.07	0.01	0.01	0.14	2	0.11	0.25	0.08	0.14	313
Follow-up^a^	Direct medical	0.69	0.38	−2.73	4.11	2	2.38	2.83	2.07	2.69	260
	Direct non-medical	0.00	–	–	–	0	0.23	1.32	0.08	0.37	22
	Indirect	0.00	–	–	–	0	0.56	4.78	0.04	1.10	25
	*Time loss [days]*	0.00	–	–	–	0	0.02	0.16	0.00	0.04	25
Total direct (OOP) cost		1.68	0.42	−2.13	5.49	**2**	5.88	9.11	4.87	6.90	313
Total economic cost		3.43	0.54	−1.41	8.25	**2**	10.08	23.11	7.52	12.66	313

*Notes*: SD, standard deviation; *n* (*c* > 0), number of caregivers with costs over zero Takas.

a Includes costs incurred by public and private healthcare facilities and providers.

Over the continuum of care for an episode of diarrhea that required hospitalization, the majority of the cost incurred was for current visit, accounting for 67%, 81%, and 81% of the total cost incurred in public, PFP, and PNFP facilities, respectively. Relative to total expenses, indirect costs from productivity loss were a significant contributor to economic losses across all types of facility and types of care, ranging from 38% to 64% (percentages are not shown in Table 5).

Table 6 shows the direct, indirect, and total costs across different characteristics of patients and caregivers. Overall, an episode of diarrhea incurred costs of about $62 for caregivers in 2018, with average OOP payments of $29 and indirect costs of $34 (Table 6). Costs were driven primarily by hospitalization and the use of private facilities. Total costs for infants under 2 years old ranged from $58 to $76, which dropped significantly to $38 for children over 2 years old (*p* = 0.000). There was a significant difference in direct costs between male and female children, with male children costing on average $30 and females $25 (*p* = 0.030. Male children were slightly more likely to be hospitalized (67% vs 62%) and were more likely to be treated in a private for-profit healthcare facility (34% vs 27%).

**Table 6:** Differences in household costs across characteristics of children and caregivers in 2018 US dollars.

Characteristic	*n*	Direct costs			Indirect costs			Total costs		
		Mean	SE	*p*-value^b^	Mean	SE	*p*-value^b^	Mean	SE	*p*-value^b^
Overall	899	28.53	1.22	–	33.74	2.12	–	62.28	3.09	-
**Age group**										
<6 months	104	26.77	3.86	0.000^c^	31.71	4.79	0.000^c^	58.48	8.41	0.000^c^
6–11 months	258	31.94	2.37		44.50	6.32		76.44	8.18	
12–24 months	404	30.18	1.87		32.06	1.86		62.24	3.51	
>24 months	133	18.31	2.35		19.56	2.41		37.88	4.50	
**Gender (child)**										
Female	322	25.41	1.89	0.019^c^	30.37	2.63	0.078^c^	55.78	4.23	0.030^c^
Male	577	30.29	1.59		35.62	2.96		65.89	4.18	
**Gender (caregiver)**										
Female	713	26.54	1.25	0.001^c^	31.93	1.74	0.036^c^	58.48	2.75	0.016^d^
Male	186	36.18	3.49		40.67	7.78		76.85	10.47	
**Study area**										
City corporation	290	52.89	2.73	0.000^c^	60.83	5.78	0.000^c^	113.71	7.83	0.000^c^
*Sylhet*	143	50.89	3.09	*0.569*^c^	49.94	4.68	*0.008*^c^	100.84	6.99	*0.435*^c^
*Rajshahi*	147	54.92	4.55		72.01	10.63		126.95	14.12	
Rural district	609	16.95	0.95		20.84	1.17		37.78	1.94	
*Maulvibazar*	302	20.48	1.65	*0.001*^c^	29.93	1.89	*0.000*^c^	50.41	3.26	*0.000*^c^
*Natore*	307	13.46	0.92		11.90	1.19		25.37	1.89	
**Residence**										
Rural	678	29.59	1.44	0.131^d^	35.23	2.67	0.182^c^	64.83	3.81	0.123^c^
Urban	221	25.29	2.30		29.15	2.60		54.44	4.57	
**Type of visit**										
Inpatient	586	40.63	1.65	0.000^c^	49.51	3.02	0.000^c^	90.16	4.25	0.000^c^
Outpatient	313	5.88	0.51		4.20	0.86		10.08	1.31	
**Facilities**										
Public	548	11.02	0.44	0.000^c^	18.07	1.01	0.000^c^	29.09	1.37	0.000^c^
PFP	348	56.18	2.43		58.54	4.96		114.72	6.78	
PNFP	3	22.16	20.48		18.63	16.90		40.79	37.38	
**Length of stay (IPD)**^a^										
<5 days	510	36.06	1.50	0.000^c^	42.90	3.15	0.000^d^	78.96	4.26	0.000^c^
≥5 days	76	71.39	6.91		93.88	8.19		165.27	13.29	
**Asset quintiles**										
Poorest	180	16.99	1.37	0.000^c^	17.17	1.52	0.000^c^	34.17	2.55	0.000^c^
2nd	181	22.08	2.12		20.30	1.81		42.38	3.56	
3rd	179	23.25	2.31		26.14	2.79		49.39	4.80	
4th	180	38.83	3.45		49.39	8.14		88.22	10.87	
Richest	179	41.60	3.39		55.83	5.11		97.44	7.77	

*Notes*: SE, standard error; *n*, number of caregivers.

a Length of stay included only hospitalized cases of diarrhea (*n* = 586).

b One-way ANOVA.

c Kruskal–Wallis rank test.

d *t*-test

Most caregivers in our sample resided and sought care in rural areas (75%) (Table 2). While caregivers residing in urban areas were more likely than rural dwellers to seek PFP healthcare for both inpatient (39% vs. 29%) and outpatient care (10% vs 6%) (Table S1), no statistical differences in direct and indirect costs were observed. The average direct costs in city corporations area ($53) was found to be significantly higher than in rural districts area ($17; *p* = 0.000).

When the lengths of stay for hospitalized patients were longer (5 days or more), direct costs ($71) were significantly higher compared with stays of less than 5 days ($36; *p* < 0.000). Indirect costs ($94) were also significantly higher for longer stays compared with stays of less than 5 days ($43; *p* < 0.000).

The direct and indirect costs were also significantly different between the poorest asset quintiles and the richest (*p* < 0.000), with the costs increasing with richer quintiles. The total cost of treatment for diarrheal cases among patients belonging to the poorest quintile was $34, compared with $97 for the richest quintile. Both direct and indirect costs were also lower among the poorest quintiles and higher among the richest quintiles.

### Economic burden

**Figure 2:**
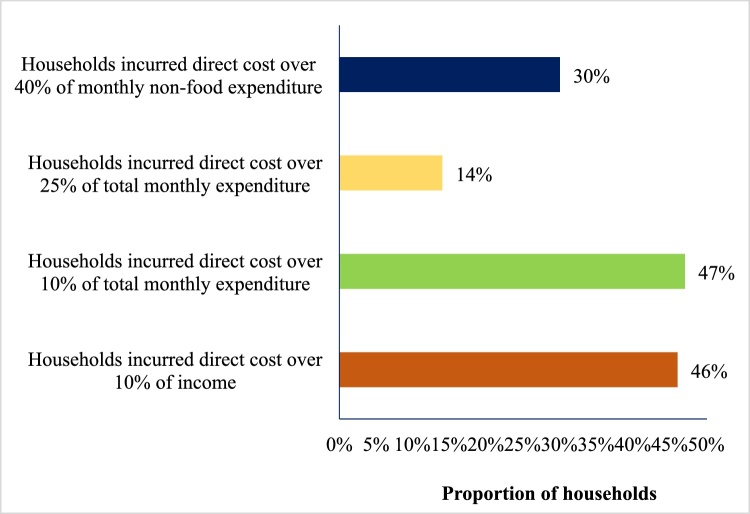
Catastrophic health expenditures related to diarrhea in Bangladesh.

Over 46% of the households had spending of over 10% of the household head’s monthly income on each episode of diarrhea. Considering the household’s consumption expenditure, 47% of the households faced CHE at the 10% threshold of household expenditures, with 14% incurring costs of over 25% of their monthly household expenditures. When excluding food, 30% of the households reported spending of over 40% of their monthly household expenditure (Figure 2).

**Figure 3:**
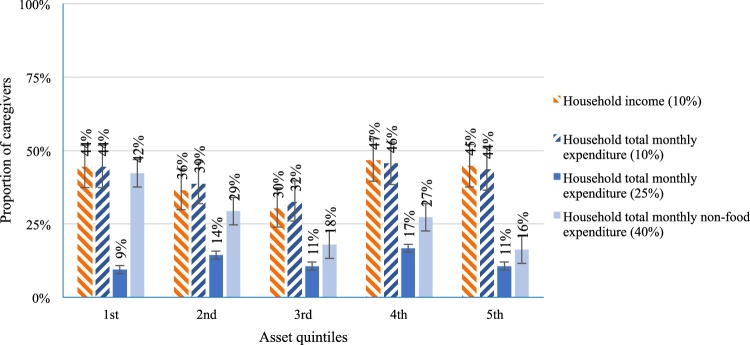
Catastrophic health expenditures related to diarrhea by asset quintile in Bangladesh.

The proportion of households that faced CHE decreased from the poorest to the richest asset quintiles. When considering consumption expenditure, excluding food, over 40% of households in the poorest asset (1st) quintile faced CHE, compared with 29%, 18%, 27%, and 16% in the 2nd, 3rd, 4th, and 5th quintiles, respectively (Figure 3).

### Societal costs and country costs of diarrhea

**Figure 4:**
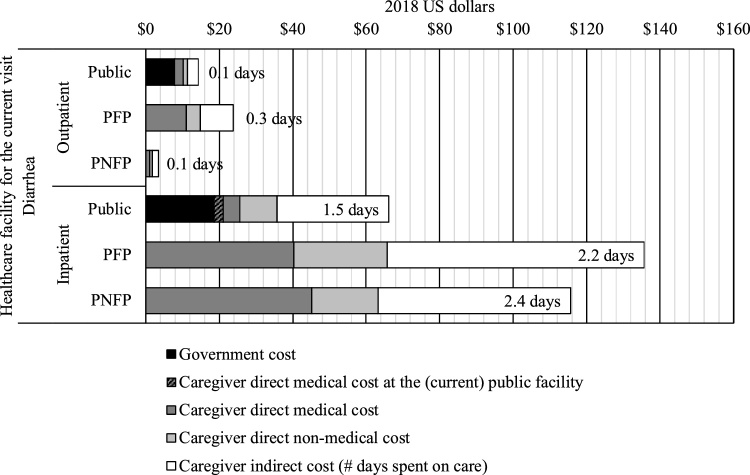
Societal costs related to diarrhea by sector in Bangladesh.

From the societal perspective, an episode of diarrhea had an average cost of $71. The societal cost for a hospitalized episode of diarrhea was $100 and that for an outpatient episode was $16. In terms of hospital types by facility ownership, the societal cost of a hospitalized episode was $66 when using public facilities and $135 when utilizing PFP facilities. For outpatient care, the lowest societal cost was associated with PNFP facilities, at $3, while public and PFP facilities followed with $14 and $24, respectively (Figure 4).

The incidence data for 2018 showed that, at a facility level, around 1 122 681 cases were reported throughout the year (DGHS, 2020). Using the average societal cost, the aggregate annual economic cost of diarrhea for 2018 in Bangladesh was estimated at approximately $79 million, or about 0.029% of 2018 GDP.

## Discussion

The average estimated societal cost per episode of diarrhea was $71, with an average cost of $100 incurred per hospitalized case and $16 per outpatient case. The government facilities spent about $23 on providing care for a hospitalized case and about $7 for an outpatient case.

In a recent study conducted in 2015, Sarker et al. estimated that the average total societal cost per episode at tertiary-level public healthcare facilities in Bangladesh was $67 (equivalent to $74 in 2018), which was similar to our estimate of $71 (Sarker et al., 2013). In another study, Sarker et al. found that caregivers spent $29 ($31 in 2018) for an episode of diarrhea, whereas for hospitalized care and outpatient care spending was $44 ($49 in 2018) and $12 ($14 in 2018), respectively. Our study found that overall caregiver costs averaged $62, which was significantly higher than those found by Sarker et al. (2013). Our higher estimate is likely due to our explicit consideration of patients from public, PFP, and PNFP facilities, while the previously mentioned study only included tertiary-level public facilities. In Bangladesh, costs for treating patients are comparatively lower in public healthcare facilities than in PFP facilities, because the public hospitals are highly subsidized (Andaleeb, 2000). Similar findings have also been observed in India, where the cost per diarrheal episode in private facilities was three times higher than for low-cost concessional hospitals and 25 times higher compared with government hospitals (Sowmyanarayanan et al., 2012). Additionally, the caregiver cost per episode at public facilities found in our study ($29) was similar to the estimates found in the India study.

Another study, conducted by Das et al., found that, on average, a household incurred $7 ($9 in 2018) for diarrheal treatment of under-5 children in rural Bangladesh. However, this study did not consider the indirect cost from productivity loss of the caregiver due to time spent treating their sick children (Das et al., 2015). Rheingans et al., in a multi-country analysis, estimated that the average household treatment cost for childhood diarrhea was $2 in Bangladesh in 2011 ($3 in 2018). The study collected information from a specific surveillance area and, as a result, had a relatively smaller sample size compared with our study (Rheingans et al., 2012b). Again, as with the other studies mentioned, the Rheingans et al. study also did not cover all types of facility where caregivers sought treatment.

In Bangladesh, OOP expenditure is a major financing mechanism for healthcare, with more than 67% of total health expenditure represented by OOP payments (MOHFW, 2017). Such a high reliance on OOP healthcare expenditure is a concern for Bangladesh due to OOP spending’s association with poverty through CHE. Moreover, our study found that caregiver costs in the poorest quintile were significantly higher than with those in the richer or richest quintiles, despite lower levels of private facility utilization. In the 1st (poorest) and 2nd quintiles, the use of private for-profit healthcare was 18% and 27%, respectively, while in the 4th and 5th (richest) quintiles, it was 54% and 65% (Supplementary Table S2). Despite lower levels of private healthcare utilization, the households in the poorest quintile faced equal or higher levels of CHE compared with households in the richest quintile (Figure 2). Similar findings have been observed in other studies (Chima et al., 2003, Sarker et al., 2013).

Our study estimated that the annual aggregate economic burden of diarrhea for treating cases of under five years of age in 2018 was $79 million. This burden would be higher if it included cases from all age groups, as found by Sarker et al., who estimated the annual economic burden of diarrheal diseases to be $172 million in 2015 ($189 million in 2018) (Sarker et al., 2018). The annual GDP per capita of Bangladesh in 2018 was $1698; based on this, an average of around 4% of per capita GDP was spent on treating diarrheal episodes (The World Bank, 2018). Moreover, the number of diarrheal admissions creates additional pressure on hospital services. Therefore, by reducing the incidence of diarrheal diseases through vaccination programs and other preventative public-health interventions, high levels of medical time use and economic costs could be averted. In addition, prevention of diarrheal illness will reduce the flow of patients to hospitals, which could save on resources allocated for the infrastructure, hospital beds, and medical staff required for management of diarrhea cases. Such saved resources could be allocated for management of other illness.

## Limitations

The limitations of this study include its cross-sectional design and being hospital based. Moreover, because of self-reported expenditures and income, it may not have captured seasonal variations in household income. However, the exit survey was conducted over a 10-month period at different facilities, which might have reduced the effects of seasonal variation. The study was not able to include the costs of emotional effects and adverse outcomes (such as tiredness and anxiety) experienced by caregivers during the treatment of diarrhea. However, this could have over/underestimated the true cost substantially, as these factors do not have market values, and may have influenced the findings of the study. Finally, the initial treatment of diarrheal disease often starts within the households; resources spent during the time prior to the initial facility visit were not fully covered in this study; however some information on costs incurred before arriving at the facility where children received care were captured.

## Conclusion

In Bangladesh, diarrhea is one of the most significant public health concerns among children under five years of age. This study found that the average economic cost for treating each episode of diarrhea was $62, amounting to around 4% of the annual national GDP per capita of 2018. More than 47% of households presenting at a facility for treatment of diarrheal disease faced a catastrophic burden of OOP expenditure due to the diarrheal episode. Therefore, any public-health interventions (e.g., vaccination) that can reduce the incidence of diarrheal diseases will have the twofold effect of improving health and alleviating poverty in Bangladesh. Data generated from this study can inform policymakers in decision making with regard to prioritization of, and investment in, diarrheal disease prevention.

## Statement on data sources

Datasets, program files and codebooks are available on open access at:

Ahmed, Sayem; de Broucker, Gatien; Hasan, Md Zahid; Mehdi, Gazi Golam; Martin del Campo, Jorge; Constenla, Dagna; Patenaude, Bryan; Uddin, Md Jasim, 2020, Cost of diarrhea in children under 5 in Bangladesh (2017–18), https://doi.org/10.7910/DVN/YKPSJ7, Harvard Dataverse, V1, UNF:6:INUNQAM/iu8oOW2iwE/Lnw== [fileUNF]

## Funding

This study was part of the Decade of Vaccine Economics (DOVE) project, funded under a multi-project grant (OPP112821) by the 10.13039/100000865Bill and Melinda Gates Foundation. The project includes empirical assessments of the cost of pneumonia, diarrhea, and measles in Bangladesh and Uganda, conducted by the International Vaccine Access Center at Johns Hopkins Bloomberg School of Public Health, the International Centre for Diarrhoeal Disease Research, Bangladesh, and Makerere University School of Public Health.

## Ethical approval

The institutional review boards of the International Diarrhoeal Disease Research Centre, Bangladesh (IRB PR-16067) and the Johns Hopkins Bloomberg School of Public Health (IRB #7256) examined the risks and benefits related to this research project and granted ethical approval.

## Conflicts of interest

Md Zahid Hasan, Gazi Golam Mehdi, Gatien de Broucker, Sayem Ahmed, Md Wazed Ali, Jorge Martin del Campo, Bryan Patenaude, Md Jasim Uddin: Received funding from the Bill and Melinda Gates Foundation to conduct this research.

Dagna Constenla: Received funding from the Bill & Melinda Gates Foundation to conduct this research. At the time of the development of this manuscript, Dr Constenla was an employee of GSK and was holding stocks as a GSK employee.
