# Supplementary material for: The economic burden of diarrhea in children under 5 years in Bangladesh
Source: Int J Infect Dis. 2021 Jun;107:37–46. doi: 10.1016/j.ijid.2021.04.038 (PMC8208894; doi:10.1016/j.ijid.2021.04.038)
Supplement: Supplementary file 1 [file mmc1.docx]

**Supplementary table**

***Table S1: Public and private healthcare use by type of care for diarrhea in Bangladesh***

|  | **Inpatient cases** | | | | | | **Outpatient cases** | | | | | |
| --- | --- | --- | --- | --- | --- | --- | --- | --- | --- | --- | --- | --- |
|  | Public | | PFP | | PNFP | | Public | | PFP | | PNFP | |
| **Age group** |  |  |  |  |  |  |  |  |  |  |  |  |
| < 6 months | 19 | 18% | 31 | 30% | 0 | 0% | 44 | 42% | 9 | 9% | 1 | 1% |
| 6–11 months | 89 | 34% | 85 | 33% | 0 | 0% | 59 | 23% | 24 | 9% | 1 | 0% |
| 12–24 months | 146 | 36% | 140 | 35% | 1 | 0% | 91 | 23% | 26 | 6% | 0 | 0% |
| > 24 months | 48 | 36% | 27 | 20% | 0 | 0% | 52 | 39% | 6 | 5% | 0 | 0% |
| **Gender (child)** |  |  |  |  |  |  |  |  |  |  |  |  |
| Female | 113 | 35% | 87 | 27% | 0 | 0% | 101 | 31% | 20 | 6% | 1 | 0% |
| Male | 189 | 33% | 196 | 34% | 1 | 0% | 145 | 25% | 45 | 8% | 1 | 0% |
| **Gender (caregiver)** |  |  |  |  |  |  |  |  |  |  |  |  |
| Female | 231 | 32% | 219 | 31% | 0 | 0% | 212 | 30% | 49 | 7% | 2 | 0% |
| Male | 71 | 38% | 64 | 34% | 1 | 1% | 34 | 18% | 16 | 9% | 0 | 0% |
| **Study area** |  |  |  |  |  |  |  |  |  |  |  |  |
| City corporation | 19 | 7% | 227 | 78% | 0 | 0% | 18 | 6% | 24 | 8% | 2 | 1% |
| *Sylhet* | 12 | 8% | 121 | 85% | 0 | 0% | 8 | 6% | 2 | 1% | 0 | 0% |
| *Rajshahi* | 7 | 5% | 106 | 72% | 0 | 0% | 10 | 7% | 22 | 15% | 2 | 1% |
| Rural district | 283 | 46% | 56 | 9% | 1 | 0% | 228 | 37% | 41 | 7% | 0 | 0% |
| *Maulvibazar* | 171 | 57% | 21 | 7% | 1 | 0% | 108 | 36% | 1 | 0% | 0 | 0% |
| *Natore* | 112 | 36% | 35 | 11% | 0 | 0% | 120 | 39% | 40 | 13% | 0 | 0% |
| **Division** |  |  |  |  |  |  |  |  |  |  |  |  |
| Sylhet | 183 | 41% | 142 | 32% | 1 | 0% | 116 | 26% | 3 | 1% | 0 | 0% |
| Rajshahi | 119 | 26% | 141 | 31% | 0 | 0% | 130 | 29% | 62 | 14% | 2 | 0% |
| **Residence** |  |  |  |  |  |  |  |  |  |  |  |  |
| Rural | 253 | 37% | 196 | 29% | 1 | 0% | 184 | 27% | 43 | 6% | 1 | 0% |
| Urban | 49 | 22% | 87 | 39% | 0 | 0% | 62 | 28% | 22 | 10% | 1 | 0% |
| **Asset quintiles** |  |  |  |  |  |  |  |  |  |  |  |  |
| 1^st^ | 87 | 48% | 25 | 14% | 0 | 0% | 60 | 33% | 7 | 4% | 1 | 1% |
| 2^nd^ | 71 | 39% | 35 | 19% | 1 | 1% | 60 | 33% | 14 | 8% | 0 | 0% |
| 3^rd^ | 65 | 36% | 41 | 23% | 0 | 0% | 61 | 34% | 12 | 7% | 0 | 0% |
| 4^th^ | 46 | 26% | 82 | 46% | 0 | 0% | 36 | 20% | 15 | 8% | 1 | 1% |
| 5^th^ | 33 | 18% | 100 | 56% | 0 | 0% | 29 | 16% | 17 | 9% | 0 | 0% |

**References**

Alam NH, Ashraf H. Treatment of infectious diarrhea in children. Pediatr Drugs 2003;5(3):151–65.

Andaleeb SS. Public and private hospitals in Bangladesh: service quality and predictors of hospital choice. Health Policy Plan 2000;15(1):95–102.

Black RE, Cousens S, Johnson HL, Lawn JE, Rudan I, Bassani DG, et al. Global, regional, and national causes of child mortality in 2008: a systematic analysis. Lancet 2008;375(9730):1969–87.

Burke RM, Smith ER, Dahl RM, Rebolledo PA, Calderón C, Cañipa B, et al. The economic burden of pediatric gastroenteritis to Bolivian families: a cross-sectional study of correlates of catastrophic cost and overall cost burden. BMC Public Health 2014;

Chima RI, Goodman CA, Mills A. The economic impact of malaria in Africa: a critical review of the evidence. Health Policy (New York) [internet] 2003;63(1):17–36. Available from: http://www.sciencedirect.com/science/article/pii/S0168851002000362

Chowdhury F, Khan IA, Patel S, Siddiq AU, Saha NC, Khan AI, et al. Diarrheal illness and healthcare seeking behavior among a population at high risk for diarrhea in Dhaka, Bangladesh. PLoS One 2015;10(6).

Das J, Das SK, Ahmed S, Ferdous F, Farzana FD, Sarker MHR, et al. Determinants of percent expenditure of household income due to childhood diarrhoea in rural Bangladesh. Epidemiol Infect 2015;143(13):2700–6.

DGHS. EPI Coverage Evaluation Survey 2013. Dhaka; 2013.

DGHS. EPI Coverage Evaluation Survey 2015. Dhaka; 2015.

DGHS. Real time health information dashboard [internet]. 2020 [cited Jun 6, 2020]. Available from: http://103.247.238.92/webportal/pages/dashboard_child_imci.php

Hoque BA, Juncker T, Sack RB, Ali M, Ashraful Aziz KM. Sustainability of a water, sanitation and hygiene education project in rural Bangladesh: a 5-year follow-up. Bull World Heal Organ 1996; 74(4)431–437 [internet]. 1996; Available from: https://apps.who.int/iris/handle/10665/54015

Hoque ME, Khan JA, Hossain SS, Gazi R, Rashid H-A, Koehlmoos TP, et al. A systematic review of economic evaluations of health and health-related interventions in Bangladesh. Cost Eff Resour Alloc 2011 Jan;9(1):12.

Jo C. Cost-of-illness studies: concepts, scopes, and methods. Clin Mol Hepatol 2014;20(4):327–37.

Khan JAM, Ahmed S, Evans TG. Catastrophic healthcare expenditure and poverty related to out-of-pocket payments for healthcare in Bangladesh — an estimation of financial risk protection of universal health coverage. Health Policy Plan [internet]. 2017;32(May):1–9. Available from: https://academic.oup.com/heapol/article-lookup/doi/10.1093/heapol/czx048

Liu L, Oza S, Hogan D, Chu Y, Perin J, Zhu J, et al. Global, regional, and national causes of under-5 mortality in 2000–15: an updated systematic analysis with implications for the Sustainable Development Goals. Lancet (London, England). 2016 Dec;388(10063):3027–35.

Mendelsohn AS, Asirvatham JR, Mkaya Mwamburi D, Sowmynarayanan T V, Malik V, Muliyil J, et al. Estimates of the economic burden of rotavirus-associated and all-cause diarrhoea in Vellore, India. Trop Med Int Heal 2008;13(7):934–42.

MOHFW. Bangladesh National Health Accounts 1997–2015. Dhaka; 2017.

MOHFW. Facility Registry: Government of People’s Republic of Bangladesh [internet]. 2020 [cited Feb 10, 2021]. Available from: http://facilityregistry.dghs.gov.bd/

O’Donnell O, van Doorslaer E, Wagstaff A, Lindelow M. Analyzing health equity using household survey data: a guide to techniques and their implementation. Washington DC: The World Bank; 2008.

Pradhan M, Prescott N. Social risk management options for medical care in Indonesia. Health Econ 2002 Jul;11(5):431–46.

Rheingans R, Kukla M, Adegbola RA, Saha D, Omore R, Breiman RF, et al. Exploring household economic impacts of childhood diarrheal illnesses in 3 African settings. Clin Infect Dis 2012a;55(Suppl 4).

Rheingans R, Kukla M, Faruque ASG, Sur D, Zaidi AKM, Nasrin D, et al. Determinants of household costs associated with childhood diarrhea in 3 South Asian settings. Clin Infect Dis 2012b;55(Suppl. 4).

Russell S. The economic burden of illness for households in developing countries: a review of studies focusing on malaria, tuberculosis, and human immunodeficiency virus/acquired immunodeficiency syndrome. Am J Trop Med Hyg 2004 Aug;71(2 Suppl):147–55.

Sack DA, Malek MA, Faruque ABUSG, Qadri F, Calderwood B, Luby SP, et al. Diarrheal epidemics in Dhaka, Bangladesh, during three consecutive floods: 1988, 1998, and 2004. Am J Trop Med Hyg 2006;74(6):1067–73.

Sarker AR, Islam Z, Khan IA, Saha A, Chowdhury F, Khan AI, et al. Cost of illness for cholera in a high risk urban area in Bangladesh: an analysis from household perspective. BMC Infect Dis [internet] 2013;13(1):518. Available from: http://bmcinfectdis.biomedcentral.com/articles/10.1186/1471-2334-13-518

Sarker AR, Sultana M, Ali N, Akram R, Alam K, Khan JAM, et al. Cost of caregivers for treating hospitalized diarrheal patients in Bangladesh. Trop Med Infect Dis 2019;4(1):1–14.

Sarker AR, Sultana M, Mahumud RA, Ali N, Huda TM, Salim M, et al. Economic costs of hospitalized diarrheal disease in Bangladesh: a societal perspective. Glob Heal Res Policy 2018;1–12.

Schroder K, Battu A, Wentworth L, Houdek J, Fashanu C, Wiwa O, et al. Increasing coverage of pediatric diarrhea treatment in high-burden countries. J Glob Health 2019;

Sowmyanarayanan T V, Patel T, Sarkar R, Broor S, Chitambar SD, Krishnan T, et al. Direct costs of hospitalization for rotavirus gastroenteritis in different health facilities in India. Indian J Med Res 2012 Jul;136(1):68–73.

The World Bank. GDP per capita (current US$) — Bangladesh | Data [Internet]. 2018 [cited Jul 4, 2020]. Available from: https://data.worldbank.org/indicator/NY.GDP.PCAP.CD?locations=BD

The World Bank. Official exchange rate (LCU per US$, period average); 2019 [Internet]. 2019 [cited Sep 9, 2020]. Available from: https://data.worldbank.org/indicator/PA.NUS.FCRF

The World Bank Group. Lower middle income | Data. 2018.

Vyas S, Kumaranayake L. Constructing socio-economic status indices: How to use principal components analysis. Health Policy Plan. 2006;21(6):459–68.

Wagstaff A, van Doorslaer E. Catastrophe and impoverishment in paying for health care: with applications to Vietnam 1993–1998. Health Econ 2003;12(11):921–34.

WHO. Generic portocols for (i) hospital-based surveillance to estimate the burden of rotavirus gastroenteritis in children and (ii) a community-based survey on utilization of health care services for gastroenteritis in children: field test version. World Health Organization; 2002.

WHO. Vaccine-preventable diseases: signs, symptoms and complications [internet]. 2012 [cited Aug 28, 2018]. Available from: http://www.euro.who.int/__data/assets/pdf_file/0005/160754/Vaccine-preventable_EN_WHO_WEB.pdf

WHO. Preventing diarrhoea through better water, sanitation and hygiene: exposures and impacts in low-and middle-income countries. World Health Organization; 2014.

Wu J, van Geen A, Ahmed KM, Alam YAJ, Culligan PJ, Escamilla V, et al. Increase in diarrheal disease associated with arsenic mitigation in Bangladesh. PLoS One 2011a;6(12):e29593.

Wu J, Yunus M, Streatfield PK, Emch M. Association of climate variability and childhood diarrhoeal disease in rural Bangladesh, 2000–2006. Epidemiol Infect 2014 Sep;142(9):1859–68.

Wu J, Yunus M, Streatfield PK, van Geen A, Escamilla V, Akita Y, et al. Impact of tubewell access and tubewell depth on childhood diarrhea in Matlab, Bangladesh. Environ Health 2011b Dec;10:109.

Xu K, Evans DB, Kadama P, Nabyonga J, Ogwal PO, Nabukhonzo P, et al. Understanding the impact of eliminating user fees: utilization and catastrophic health expenditures in Uganda. Soc Sci Med 2006 Feb;62(4):866–76.

Xu K, Evans DB, Kawabata K, Zeramdini R, Klavus J, Murray CJL. Household catastrophic health expenditure: a multicountry analysis. Lancet 2003 Jul;362(9378):111–7.
